# Impact of recording length and other arrhythmias on atrial fibrillation detection from wrist photoplethysmogram using smartwatches

**DOI:** 10.1038/s41598-022-09181-1

**Published:** 2022-03-30

**Authors:** Min-Tsun Liao, Chih-Chieh Yu, Lian-Yu Lin, Ke-Han Pan, Tsung-Hsien Tsai, Yu-Chun Wu, Yen-Bin Liu

**Affiliations:** 1 Division of Cardiology, Department of Internal Medicine, National Taiwan University Hospital Hsinchu Branch, Hsinchu, Taiwan; 2 Department of Medicine, College of Medicine, National Taiwan University, Taipei, Taiwan; 3 Present Address: Division of Cardiology, Department of Internal Medicine, National Taiwan University Hospital, No.7, Zhongshan S. Rd., Zhongzheng Dist., Taipei City, 100 Taiwan; 4 Value Lab, Acer Inc., New Taipei City, Taiwan

**Keywords:** Cardiology, Medical research, Engineering

## Abstract

This study aimed to evaluate whether quantitative analysis of wrist photoplethysmography (PPG) could detect atrial fibrillation (AF). Continuous electrocardiograms recorded using an electrophysiology recording system and PPG obtained using a wrist-worn smartwatch were simultaneously collected from patients undergoing catheter ablation or electrical cardioversion. PPG features were extracted from 10, 25, 40, and 80 heartbeats of the split segments. Machine learning with a support vector machine and random forest approach were used to detect AF. A total of 116 patients were evaluated. We annotated > 117 h of PPG. A total of 6475 and 3957 segments of 25-beat pulse-to-pulse intervals (PPIs) were annotated as AF and sinus rhythm, respectively. The accuracy of the 25 PPIs yielded a test area under the receiver operating characteristic curve (AUC) of 0.9676, which was significantly better than the AUC for the 10 PPIs (0.9453; P < .001). PPGs obtained from another 38 patients with frequent premature ventricular/atrial complexes (PVCs/PACs) were used to evaluate the impact of other arrhythmias on diagnostic accuracy. The new AF detection algorithm achieved an AUC of 0.9680. The appropriate data length of PPG for optimizing the PPG analytics program was 25 heartbeats. Algorithm modification using a machine learning approach shows robustness to PVCs/PACs.

## Introduction

Atrial fibrillation (AF) is currently the most common type of arrhythmia, with a lifetime incidence of approximately 20–25% among Caucasian and Oriental populations^1–3^. AF is associated with an increased long-term risk of stroke, heart failure, and all-cause mortality^1^. The diagnosis of AF relies mainly on clinical symptoms and a short-period electrocardiogram (ECG) examination^3^. AF is defined as paroxysmal if it lasts < 7 days and persistent if it lasts ≥ 7 days^3^. Although both paroxysmal and persistent AF are associated with a similar risk of stroke, paroxysmal AF is more difficult to detect using a short-period ECG because of its paroxysmal nature^4^. Furthermore, approximately one-third of the patients with AF are asymptomatic^5^. Therefore, continuous monitoring and frequent ECG recordings can increase the AF detection rate. However, ECG-based screening strategies for AF may yield great heterogeneity among studies due to various monitoring durations and frequencies ^6^. Although implantable cardiac monitoring can effectively increase the diagnostic rate of AF^7^, the high cost and invasive nature of the procedure limits its use to a relatively healthy population.

Photoplethysmography (PPG) is an optical technology that can detect changes in blood flow. The PPG principle was first proposed in 1937 by Hertzman^8^. When the skin is irradiated with light, it absorbs a part of the light’s intensity. The intensity of the reflected light can then be measured, and it correlates with the volume of blood supplied to the tissue. Cyclic changes in systolic and diastolic blood pressures cause similar cyclic changes in the blood volume. Therefore, the cycle length of the heartbeat can be determined based on the reflected light intensity. The PPG signal waveform indicates a change in the blood volume produced by each heartbeat. PPG is used in AF detection algorithms, and it allows passive and continuous monitoring using modern wearable devices^9,10^. PPG has emerged as a low-cost and non-intrusive modality for continuous heart rate monitoring^11^. Thus, PPG may be an attractive alternative to the existing ECG-based solutions for AF detection. Currently, algorithms exist for automatic AF detection using PPG technology^11^. If such technology can be translated into smartwatch devices, it could become increasingly affordable and accessible to the general public. To be useful, a PPG-based AF detection algorithm for smartwatches should have low computational cost and low memory requirements^12^. A validated PPG-based AF detection algorithm with a high degree of accuracy using only PPG and accelerometer data from a wrist-worn smartwatch would ensure superiority in AF diagnosis^13–15^.

In this study, we prospectively obtained continuous ECG waveforms (using intracardiac electrogram recordings in the majority of cases) recorded using an electrophysiology recording system and PPG signals recorded using a wrist-worn smartwatch simultaneously in patients undergoing AF catheter ablation, either by radiofrequency or cryotherapy or electrical cardioversion for AF. Both AF and sinus rhythm (SR) were recorded during the procedure. PPGs from patients with frequent premature ventricular/atrial complexes (PVCs/PACs) who underwent catheter ablation were also used to evaluate the impact of other arrhythmias on the accuracy of the AF detection algorithm.

## Results

### Study population

Our study sample included 116 patients, 38 women (33%), with a mean age of 59.6 ± 11.4 years and an average CHA2DS2-VASc score of 1.9 ± 1.5. Of the 116 patients, 76 were diagnosed with paroxysmal AF, and 40 with persistent AF. Baseline characteristics of the study groups are presented in Table 1.

**Table 1:** Baseline characteristics of study participants.

**Clinical characteristics**	n = 116
Age, years	59.6 ± 11.4
Male	78 (67.2)
BW, kg	72.3 ± 13.3
BH, cm	165.4 ± 9.2
BMI, kg/m^2^	26.3 ± 3.6
Smoking	34 (29.3)
CHADS2-Vasc score	1.9 ± 1.5
Congestive heart failure	15 (12.9)
**NYHA Fc**	
I	6 (5.2)
II	6 (5.2)
III	3 (2.6)
IV	0
Hypertension	60 (51.7)
Diabetes mellitus	29 (25.0)
Cerebral vascular accidence/TIA	11 (9.5)
Coronary artery disease	8 (6.9)
Peripheral artery disease	0
Chronic kidney disease	12 (10.3)
End stage renal disease	3 (2.6)
**Classification of atrial fibrillation**	
Paroxysmal atrial fibrillation	76 (65)
Persistent atrial fibrillation	40 (35)
**Type of procedure**	
Radiofrequency catheter ablation	82 (70.7)
Cryoballoon ablation	22 (19.0)
Cardioversion	12 (10.3)
**Pharmacologic therapy**	
Beta-blockers	78 (67.2)
ACEi/ARB	46 (39.7)
Calcium channel blockers	27 (23.3)
Diuretics	30 (25.9)
Amiodarone	68 (58.6)
Propafenone	47 (40.5)
Dronedarone	3 (2.6)
Sotalol	4 (3.4)
Flecainide	11 (9.5)
Digoxin	3 (2.6)

### Extracted PPG features

Of the recorded PPG signals, 18% were labeled as unreliable due to the following conditions: (1) If the observed heartbeat was > 200 bpm at PPG sample rate 512 Hz (depending on device), the observed PPI was calculated by 60/200*512. This study excluded PPIs that were less than 60/200*512; (2) the smoothed PPG signal with a wide range FFT spectrum was excluded because it was noise. A ratio was obtained by dividing the sum of the FFT spectrum at > 30 Hz (depending on the device) by the total sum of the FFT spectrum. A smoothed PPG signal was removed if this ratio was > 0.5; and (3) the smoothed PPG signal with an extremely sharp peak was excluded. A smoothed PPG signal with a sharp peak is defined as max_peak_ height/median _peak_height $\ge$ 4.5, IQR_peak_ height/median _peak_height $\ge$ 2.15, and mean_peak_height/median_peak_height $\ge$ 1.3. These thresholds were selected via k-means clustering using the above ratios. After deleting these signals, 6475 and 3957 of the 25-beat PPG segments were labeled AF and SR, respectively. In the univariate analysis, most PPG features were significantly different between the AF and SR signals, including the time domain and frequency domain analyses of PPI, peak height analysis of PPG, and ACF features of PPI (Table 2).

**Table 2:** Univariate analysis of PPG features of 25-beat PPI segments between AF and SR PPI.

Features	AF PPI (N = 6475)	SR PPI (N = 3957)	*P* value
**Time domain**			
PPI SD	$0.2039\pm 0.0891$	$0.0910\pm 0.1082$	$<.001$
PPI RMSSD	$0.2881\pm 0.1267$	$0.1244\pm 0.1446$	$<.001$
PPI Entropy (bins = 10)	$1.8340\pm 0.2590$	$1.5594\pm 0.6206$	$<.001$
PPI Entropy (bins = 100)	$2.9338\pm 0.1522$	$2.5852\pm 0.3570$	$<.001$
PPI Entropy (bins = 1000)	$3.1070\pm 0.0889$	$2.8164\pm 0.2097$	$<.001$
Rolling PPI SD3	$0.2092\pm 0.0725$	$0.0889\pm 0.0766$	$<.001$
Rolling PPI RMSSD3	$0.0691\pm 0.0554$	$0.0857\pm 0.0835$	$<.001$
**Frequency domain of PPI**			
MaxFFT	$1.2431\pm 0.3512$	$1.2457\pm 0.2998$	$0.6867$
Rolling MaxFFTSD3	$0.2021\pm 0.1602$	$0.0345\pm 0.0832$	$<.001$
**Peak height**			
Peak height SD	$0.3287\pm 0.1111$	$0.1975\pm 0.1562$	$<.001$
Peak height RMSSD	$0.4745\pm 0.1618$	$0.2651\pm 0.2520$	$<.001$
Peak height IQR	$0.4086\pm 0.1685$	$0.2342\pm 0.2496$	$<.001$
**ACF Feature of PPI**			
ACF peak height SD	$0.1458\pm 0.1019$	$0.1752\pm 0.1347$	$<.001$
ACF peak height RMSSD	$0.1935\pm 0.1494$	$0.2143\pm 0.2093$	$<.001$
ACF peak height IQR	$0.1658\pm 0.1230$	$0.2122\pm 0.1721$	$<.001$
ACF peak height Diff SD	$0.1926\pm 0.1569$	$0.2178\pm 0.2370$	$<.001$

Within-patient differences in the PPG features were also examined (Table 3). The time and frequency-domain analyses of PPIs and peak height analyses of the PPG features were significantly different between AF and SR. Although MaxFFT is an estimate of heart rate, the SD of MaxFFT within the three PPI segments is advantageous as it provides a measure of heart rate variability.

**Table 3:** Univariate analysis of the within-subject mean difference between the average of PPG features of 25-beat PPI segments for AF and SR.

Features	AF-SR (N = 74)	95% CI	*P* value
**Time domain**			
PPI SD	$0.1017\pm 0.0864$	$\left(\mathrm{0.0817,0.1218}\right)$	$<.001$
PPI RMSSD	$0.1416\pm 0.1179$	$\left(\mathrm{0.1143,0.1690}\right)$	$<.001$
PPI Entropy (bins = 10)	$0.2633\pm 0.3112$	$\left(\mathrm{0.1912,0.3354}\right)$	$<.001$
PPI Entropy (bins = 100)	$0.3032\pm 0.2250$	$\left(\mathrm{0.2511,0.3553}\right)$	$<.001$
PPI Entropy (bins = 1000)	$0.2389\pm 0.1469$	$\left(\mathrm{0.2049,0.2730}\right)$	$<.001$
Rolling PPISD MA3	$0.1064\pm 0.0911$	$\left(\mathrm{0.0848,0.1280}\right)$	$<.001$
Rolling PPIRMSSD SD3	$-0.0226\pm 0.0468$	$\left(-0.0336,-0.01148\right)$	$<.001$
**Frequency domain of PPI**			
Max FFT freq	$0.0724\pm 0.2925$	$\left(\mathrm{0.0047,0.1402}\right)$	$0.036$
Rolling max FFT Freq SD3	$0.1858\pm 0.1363$	$\left(\mathrm{0.1535,0.2180}\right)$	$<.001$
**Peak height**			
Peak height SD	$0.1244\pm 0.1328$	$\left(\mathrm{0.0936,0.1551}\right)$	$<.001$
Peak height RMSSD	$0.1985\pm 0.1954$	$\left(\mathrm{0.1533,0.2438}\right)$	$<.001$
Peak height IQR	$0.1716\pm 0.2136$	$\left(\mathrm{0.1221,0.2211}\right)$	$<.001$
**ACF feature of PPI**			
ACF peak height SD	$-0.0181\pm 0.0914$	$\left(-\mathrm{0.0393,0.0031}\right)$	$0.09287$
ACF peak height RMSSD	$-0.0168\pm 0.1431$	$\left(-\mathrm{0.0499,0.0163}\right)$	$0.3157$
ACF peak height IQR	$-0.0332\pm 0.1150$	$\left(-0.0598,-0.0065\right)$	$0.01535$
ACF peak height Diff SD	$-0.0329\pm 0.1744$	$\left(-\mathrm{0.0733,0.0075}\right)$	$0.1089$

### Optimal heartbeats and ROC curves for AF detection

**Figure 1:**
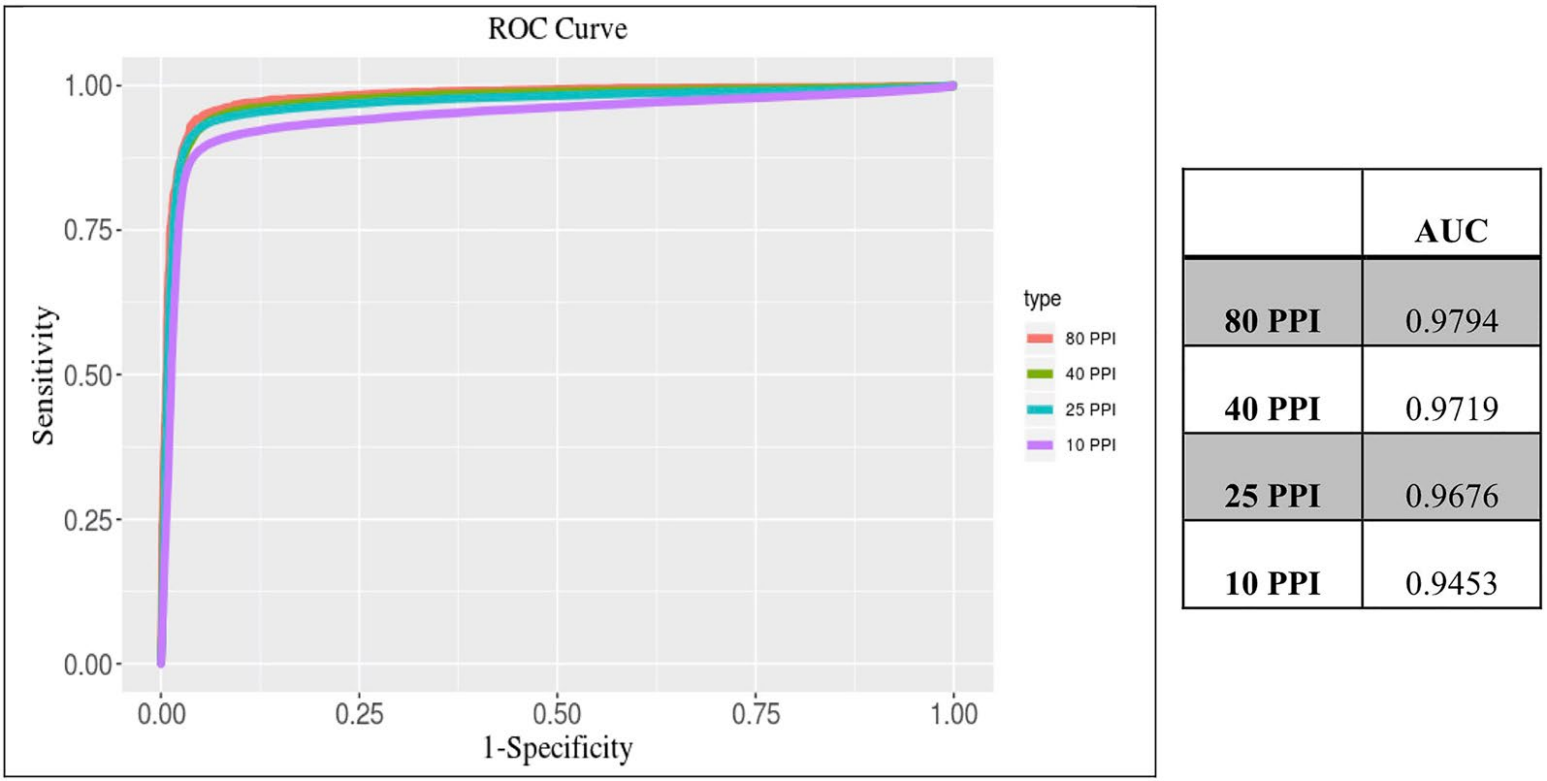
Receiver operator characteristic (ROC) curves of photoplethysmogram (PPG) models including the six independent PPG features extracted from the 10, 25, 40, and 80-beats of the pulse-to-pulse interval to detect AF.

The ROC curves of our PPG analytics program, which included eight features—PPI SD, RMSSD, Shannon entropy (SE10, SE100, and SE1000), rolling SD3, RMSSD3, and MaxFFTSD3 for AF discrimination—constructed using the 10, 25, 40, and 80 heartbeat data are shown in Fig. 1.

The accuracy of all eight PPG features determined from the 25-beat PPI (AUC, 0.9676; 95% confidence interval (CI) 0.9656–0.9697) was better than that of the 10-beat PPI (AUC, 0.9453; 95% CI 0.9437–0.9469; *P* < 0.001). After the first analysis, we decreased the sampling rate of the PPI to 128 Hz, which is acceptable for commercial use, and found that the 95% CI for the 25-beat PPI intersected the 95% CI for the 25-beat PPI recorded at a sampling rate of 512 Hz. Therefore, the accuracy of the parameters obtained from the signals sampled at 512 Hz was not significantly different from those at 128 Hz (AUC, 0.9676 versus 0.9643, respectively). Finally, the sensitivity, specificity, positive predictive value, negative predictive value, and accuracy of AF discrimination of the 25-beat PPG analytics program were 96%, 92.5%, 95.6%, 94.7%, and 95.2%, respectively (Table 4). Although the 40- and 80-beat programs have larger AUC values (0.9719 and 0.9794, respectively), the longer recording duration and increased sensitivity to movement artifacts would attenuate their application in ambulatory clinical settings.

**Table 4:** Sensitivity, specificity, positive predictive value, negative predictive value, and accuracy among different patients between the 10-beat, 25-beat, 40-beat, and 80-beat analytic programs.

PPIs	AUC	AUC 95% CI	Accuracy (%)	Sensitivity (%)	Specificity (%)	PPV (%)	NPV (%)
10	0.9453	0.9437–0.9469	93.65	93.05	95.44	94.06	93.05
25	0.9676	0.9656–0.9697	95.29	96.05	92.50	95.63	94.73
40	0.9719	0.9695–0.9743	95.22	96.57	92.90	95.93	94.14
80	0.9794	0.9761–0.9826	95.93	97.03	93.81	96.82	94.37

### Optimal heartbeats and AUC for within-subject AF detection

To determine if the patient-specific algorithm could be improved, we collected earlier data points for both AF and SR of each patient to run the model training procedure and feature extraction as previously described. The remaining data points with a late timestamp were used to validate the accuracy of the model. The accuracy of all eight PPG features determined from the 25-beat program was better than those obtained from other beat programs. The AUC was 0.9716 (95% CI 0.9663–0.9770) for the 25-beat program versus 0.9518 (95% CI 0.9480–0.9557) for the 10-beat program (*P* < 0.001), while the AUC of the 40- and 80-beat programs decreased to 0.9652 and 0.9336, respectively. This decrease may have resulted from fewer PPI segments (corrupted by motion artifacts and noise signals) available to train the 40- and 80-beat SVM programs. The sensitivity, specificity, positive predictive value, negative predictive value, and accuracy of AF discrimination for the 25-beat PPG analytic program were 97%, 94%, 96%, 95.4%, and 95.8%, respectively (Table 5). Based on these results, we determined that the 25-beat program was optimal in an ambulatory clinical environment.

**Table 5:** Sensitivity, specificity, positive predictive value, negative predictive value, and accuracy in each patient between 10-beat, 25-beat, 40-beat, and 80-beat analytic programs.

PPIs	AUC	AUC 95% CI	Accuracy (%)	Sensitivity (%)	Specificity (%)	PPV (%)	NPV (%)
10	0.9518	0.9480–0.9557	92.72	93.22	91.95	94.57	90.02
25	0.9716	0.9663–0.9770	95.85	97.03	94.07	96.09	95.47
40	0.9652	0.9575–0.9730	95.67	97.17	93.44	95.65	95.70
80	0.9336	0.9161–0.9510	91.72	93.71	88.79	92.44	90.62

### Premature ventricular and atrial complexes for further analysis

We included the PVC/PAC data recorded by 24-h Holter ECG from 36 patients [mean age = 56.0 ± 15.3 years] with frequent PVCs, and a mean PVC burden of 22.2 ± 13.6% and two patients [mean age = 54 years] with frequent PACs and a mean PAC burden of 12.7%. We prospectively collected continuous waveforms of ECG and PPG signals simultaneously in patients undergoing radiofrequency treatment for frequent PVCs or PACs. A total of 3210 segments of 25-beat PPIs of PPG were labeled as PVCs, and a total of 111 segments of 25-beat PPIs of PPG were labeled as PACs. We labeled the previous 3957 segments of the SR, 3210 segments of the PVCs, and 111 segments of the PACs as non-AF. All segments, including 6475 segments of 25-beat PPIs of AF and 7278 segments of 25-beat PPIs of non-AF, were tested in our AF detection model.

The accuracy of the original method was an AUC of 0.9263 (95% CI 0.9213–0.9312) for the 25-beat program when PPG segments with PVC/PAC were included. The presence of PVCs/PACs compromised AF detection accuracy. After radial basis function (rbf) kernel adjustment, the AUC increased to 0.9610 (95% CI 0.9577–0.9642). When we added three additional features (Lag1ACF and Lag2ACF, the p-value of the unimodality test, even and odd PPI SDs) to the rbf kernel, the AUC increased to 0.9629 (95% CI 0.9597–0.9660). Furthermore, when the model was changed to a random forest model, the AUC increased to 0.9680 (95% CI 0.9651–0.9710). Finally, the sensitivity, specificity, positive predictive value, negative predictive value, and accuracy of AF discrimination for the 25-beat PPG analytic final program was 94.1%, 93.4%, 93%, 93.9%, and 93.7%, respectively (Table 6).

**Table 6:** Sensitivity, specificity, positive predictive value, negative predictive value, and accuracy between (A) original model, (B) original model with rbs kernel adjustment, (C) model B with three additional features, and (D) features in model C with a random forests approach.

PPIs	AUC	AUC 95% CI	Accuracy (%)	Sensitivity (%)	Specificity (%)	PPV (%)	NPV (%)
A	0.9263	0.9213–0.9312	90.63	93.03	88.50	87.81	92.86
B	0.9610	0.9577–0.9642	92.17	92.89	91.44	90.88	92.96
C	0.9629	0.9597–0.9660	92.24	92.17	92.30	91.70	92.32
D	0.9680	0.9651–0.9710	93.76	94.11	93.46	93.09	93.99

## Discussion

The main findings of this study were as follows: (1) quantitative analysis of PPG waveforms recorded using a consumer wrist-worn smartwatch can clearly discriminate signals of AF from the background SR with a within-subject AUC of 0.972 and between-subject AUC of 0.968; (2) the appropriate data length of PPG to optimize the PPG analytics program was 25 heartbeats; (3) frequent PVCs/PACs reduced the accuracy of the AF detection algorithm, and algorithm optimization could achieve an AUC of 0.9680; and (4) the combination of a noise reduction algorithm with triaxial ACC data and short-duration PPG analysis (< 15 s) could lead to the development of ambulatory diagnostic tools for early detection of AF in the general public who are at risk of developing AF.

PPGs can be collected on most modern wearable devices and are often monitored both passively and continuously. Hence, a PPG-based AF algorithm requires less active effort from the participant, increasing the ease of adoption and potential for AF diagnosis among individuals with paroxysmal AF and asymptomatic individuals. Several recent studies have utilized PPG readings measured via smartphones^16^, and algorithms tailored to existing wrist-worn devices have been developed; however, this technology remains limited. This is due to the highly variable signal quality of the PPG on many wearable devices, which is caused by background noise, the variable quality of sensor technologies in the devices, and motion artifacts that result from individual user activity.

For these reasons, no algorithm has been developed that can be deployed to existing consumer wearable technologies while maintaining performance levels on par with ECG–based approaches^17^. PPG triggered on-demand wearable ECGs can be used for long-term AF surveillance and management^18^. Wearable PPG is more suitable for large-scale screening of AF in asymptomatic participants at risk for stroke, as well as monitoring of AF burden, which is associated with the symptoms of AF patients, whereas wearable ECG is more specific for the confirmation of AF diagnosis^19^.

The quality of PPG signals is a major challenge in the development of arrhythmia detection methods. In general, PPG signals recorded from a wrist-worn device have a lower signal/noise ratio than those recorded from fingertip devices. Several studies have addressed the PPG signal quality for AF detection. Solosenko et al.^20^ reported a sensitivity of 72% and specificity of 99.7% when 89.2% of the database was available for analysis and a sensitivity of 97.2% and specificity of 99.6% when the demand for signal quality was increased, such that only 50% of the data were available for analysis. This research illustrated the impact of PPG signal quality on the accuracy of the AF detection method.

Deep learning approaches for PPG signal quality assessment have been proposed to achieve higher performance for AF detection by increasing the robustness to noise ratio and motion artifacts inherent to PPG signals^21–23^. However, the intensive requirement of computational power may limit its use as consumer wearable devices. In this study, we added ACC function. We noted that if the motion intensity is too high, the accuracy of AF detection will be markedly reduced owing to the noise and unstable baseline of the PPG signals. Our results showed that with the addition of ACC function and confirmation of a quiet patient state, a 25-beat program was ideal for detecting AF. This two-step method has also been used in previous studies, which can effectively remove the interference caused by motion artifacts, thereby providing high-quality PPG signals^22,23^.

This study investigated whether a validated PPG-based AF detection algorithm with a high degree of accuracy using only PPG and ACC data from a wrist-worn smartwatch could ensure superiority in AF diagnosis. PPG obtained using a smartwatch offers the added benefit of long-term continuous monitoring and potentially increases the sensitivity of AF detection. However, wearable devices have limited energy and constraints on battery size and heat dissipation. Owing to these limitations, PPG-based AF algorithms can be combined with smartphone applications. Although some computation-intensive algorithms, such as deep learning models, can work on a remote server with powerful processors, they may not be suitable for a smartphone’s microprocessor. In this study, we used a machine learning-based SVM approach for AF detection. The computational requirements for the algorithm that we used can be embedded in consumer smartphones, providing a short-latency output for real-time realizability.

The current European guidelines for the management of AF suggest opportunistic screening in patients older than 65 years using pulse checks or single-lead portable ECG devices (Class I recommendation)^3^. In contrast, a subsequent finding suggested that ECG-based screening does not improve the detection rate of asymptomatic AF compared with the pulse palpation-based approach, and there is currently no evidence to support the benefits of ECG-based screening^24^. The CRYSTAL-AF trial randomized 441 patients with cryptogenic stroke without a history of AF and used an implantable cardiac monitor (ICM) versus standard care to monitor the occurrence of AF. The control and ICM groups included 220 and 221 patients respectively, and the AF detection rate was significantly higher in the ICM group, both at 6 and 12 months (hazard ratio [HR] 6.4, 95% CI 1.9–21.7; HR 7.3, 95% CI 2.6–20.8, respectively). With the extension of monitoring time in the ICM group, the rate of diagnosis continued to increase through 1, 6, 12, and 36 months (3.7%, 8.9%, 12.4%, and 30%, respectively)^25^. This study revealed that the longer the duration of monitoring, the higher the detection rate of AF. However, implanting an ICM is an invasive and expensive procedure, thereby limiting the utilization of this technology for the general public. Therefore, finding a low-cost and non-intrusive modality is necessary for AF screening. The newest generation of smartwatches provide continuous heart rate monitoring via PPG, and there are some automated algorithms for atrial fibrillation detection in the literature that use similar technologies^26,27^.

Our study identified the distinguishing PPG features of AF and investigated the optimal heartbeat program for PPG data collection. Four different heartbeat programs for PPG (10, 25, 40, and 80 heartbeats) were investigated. Several AF detection methods collect PPG data for a fixed duration^11,20,21^. A disadvantage of these methods is that the sample size of PPI in a fixed duration depends on the heart rate, and differences in the sample size influence the value of the features. Consideration of a fixed sample size of PPI for AF detection results in a dynamic data collection duration: shorter data collection time for a higher heart rate. In this study, we showed that a longer PPI length in one segment improved the accuracy of AF detection. However, in clinical applications, a shorter duration of signal sampling for AF detection is preferable. In this regard, we note that single-segment features with N heartbeats (SD, RMSSD, and Shannon entropy) were useful in estimating the population distribution f(x) and variance ${\sigma }^{2}$. For example, SD^2^ is an unbiased estimator of ${\sigma }^{2}$, with a larger N, improving the estimation of the real parameters. Our problem was how large of an N is sufficient to ensure that these statistics can distinguish between AF and SR. We determined that ten PPIs provided insufficient data to reliably estimate the distribution parameter, resulting in a low AUC. In contrast, the inclusion of 25 heartbeats optimized the output of the PPG analytics program. Meanwhile, a longer PPI segment was more sensitive to motion artifacts, limiting the data available for analysis based on our method. The 25-beat segment provides a balance between diagnostic accuracy and recording duration.

Instead of PPG duration, the number of PPIs was considered the variable of interest because we treated PPI as a random variable with a probability distribution. We assumed a PPI distribution f(x) with μ and ${\sigma }^{2}$, satisfying the condition that ${\sigma }^{2}$ is not a function of μ. As such, SD, RMSSD, and Shannon entropy are heart-rate-robust features. SD, RMSSD, and Shannon entropy are also “sufficient” to retain information about variance. Instead of collecting 3 N PPI samples continuously, we extracted features from each segment with sample size N and used the average of the three-segment features. This method shortened the data collection time and aggregated more data to maintain accuracy. Thus, all eight features extracted to distinguish AF from SR in our study were heart-rate-robust features and required a shorter recording time for AF detection when the heart rate was higher during AF.

Many studies have been conducted on AF detection using wrist PPG sensors; however, the data collected for algorithm development were relatively pure AF and SR data^12–15^. In the general population, many other arrhythmias cause irregular pulses, such as multifocal atrial tachycardia, ectopic atrial rhythm, and PVCs. The presence of other arrhythmias within a recording poses a challenge for AF detection, as other arrhythmias may show mixed characteristics from the distributions of both AF and SR. Bashar et al. used a two-step detection method with PAC/PVC pattern recognition to reduce the false-positive rate of AF detection^28^. In our study, after adding PVC and PAC data, the AUC of the original AF detection algorithm reduced from 0.9676 to 0.9263. Therefore, we analyzed the addition of features and changed the SVM to an RF model. Our results showed that algorithm modification using a machine-learning approach achieved an AUC of 0.9680 and showed robustness in PVCs/PACs. This new algorithm is expected to be better at discriminating between AF and AF-mimicking rhythms, leading to higher clinical-grade performance.

In clinical settings, if such a PPG algorithm can be validated and integrated into a smartwatch device, it may become an important tool for clinical screening for AF in the general population as well as a more convenient and cheaper method for prolonged monitoring of AF in patients with a high cardiovascular risk. In this study, we investigated the best analytical PPG algorithm and the most appropriate PPG data recording duration for AF detection. Our results may further the development of smartwatch software for ambulatory patients. Ultimately, improved detection of silent subclinical AF may have clinical significance in terms of stroke prevention using prophylactic anticoagulants.

Recently, a large-scale study including more than 40,000 participants demonstrated a low probability of receiving an irregular pulse notification, with 84% of the notifications concordant with AF^26^. Therefore, a prediagnosis notification should be triggered by a validated PPG-based AF detection algorithm, and the subsequent single-lead ECG recording can be used as a confirmation tool.

A continuous wearable PPG monitor can be used to estimate the AF burden. A higher AF burden is associated with a higher risk of stroke, prevalence and incidence of heart failure, and risk of mortality^29^. Developments in monitoring technologies will likely change the landscape of long-term AF management and could better define the clinical significance of alterations in the AF burden over time.

There may be some false notifications when monitoring healthy individuals using a PPG algorithm because the pre-test probability of AF in such individuals is lower than that of high-risk patients. Our findings have shown that an ideal PPG analytics program can still give rise to misclassification as motion artifacts, and environmental noise may not be completely removed. This may cause misjudgment during signal processing. The PPI obtained may differ from the actual PPI. However, some PVCs/PACs, sinus arrhythmia, and short atrial runs under the baseline SR can produce relatively irregular PPIs, which causes the program to misjudge the SR as AF. In contrast, if the PPI of AF is relatively regular with fewer variations, it may cause the program to misjudge AF as SR. Therefore, the optimization of the program should be investigated.

This study had some limitations. First, this study only included patients who underwent AF catheter ablation or cardioversion. Therefore, the generalizability of these findings to relatively low-risk patients requires further research. However, the data represents the ECG and PPG of AF and SR in the same patients. The detected PPG and ECG signals were simultaneously obtained, which may be feasible for the general population. Second, despite our results showing a better analysis method for discriminating AF from non-AF, including SR, PVCs, and PACs using a machine learning approach, larger training databases are needed to develop an algorithm to discriminate between SR, AF, and other arrhythmias. Third, our ROC curves were created using only the data remaining after excluding 18% of unreliable signals. PPG signals from patients with moving wrists are undoubtedly noisy. It is challenging to analyze low-quality PPG signals. Therefore, our research focuses on high-quality PPG signals with an accurate discrimination ability. Finally, we did not analyze whether other patient-specific clinical parameters, such as age and sex, affected the accuracy of interpretation. However, the purpose of our study was to apply the PPG analytics program to detect the occurrence of AF without considering various clinical parameters.

To conclude, quantitative analysis of PPG waveforms recorded using a wristwatch can clearly discriminate AF signals from those of the SR. The appropriate data length of the PPG for optimizing the PPG analytics program was 25 heartbeats.

## Methods

### Study design and population

This study was conducted at the electrophysiology laboratory of the National Taiwan University Hospital. Patients who were admitted to our institution for AF catheter ablation either by radiofrequency or cryotherapy, or electrical cardioversion for the treatment of symptomatic AF refractory to antiarrhythmic drugs between January 2018 and January 2020 were prospectively enrolled. To evaluate the impact of other arrhythmias on AF detection, patients admitted to our institution for PVC/PAC catheter ablation between September 2018 and January 2021 were prospectively enrolled. Written informed consent was obtained from all patients, and the study was approved by the Institutional Review Board of the National Taiwan University Hospital (approved protocol codes 201804006RIPB, 20180507 and 201909044RIPC, 20191101). All methods were performed in accordance with the relevant guidelines and regulations. The indications for catheter ablation were based on the latest guidelines^30^. The inclusion criteria were as follows: (1) indications for AF/PVC/PAC catheter ablation or AF cardioversion, (2) age ≥ 18 years, and (3) capacity and willingness to provide informed consent for participation in the study. The exclusion criteria were as follows: (1) enrolment in other clinical studies, (2) pregnancy, and (3) mental disorders. After patients consented to participate in the study, their demographic data were prospectively collected.

### ECG and PPG data

Continuous ECG waveforms obtained using an electrophysiology recording system (LabSystem PRO; Boston Scientific, Boston, MA, USA) and PPG signals using a wrist-worn smartwatch were simultaneously collected from patients undergoing radiofrequency or cryotherapy ablation for AF. Both AF and SR were recorded during the AF ablation or cardioversion procedures. Wrist-worn smartwatches (Leap Ware Smartwatch; Acer Inc., Taiwan) with green-light PPG sensors and triaxial accelerometers were used to record PPG and accelerometer (ACC) signals, respectively. The sampling rates were 100 Hz for the ACC signals and 512 Hz for the PPG signals. A higher sampling rate of 512 Hz for PPG was initially used to determine if the morphological features of the PPG signal were helpful in distinguishing AF from SR. Data for both AF and SR were continuously measured for at least 20 min. Two authors of this study, who are electrophysiologists, reviewed and noted the start and end times of AF and SR. AF was defined as an irregular rhythm if > 30 s in duration, without a clearly fixed P-wave before the QRS complex^3^.

### Proposed detection framework

**Figure 2:**
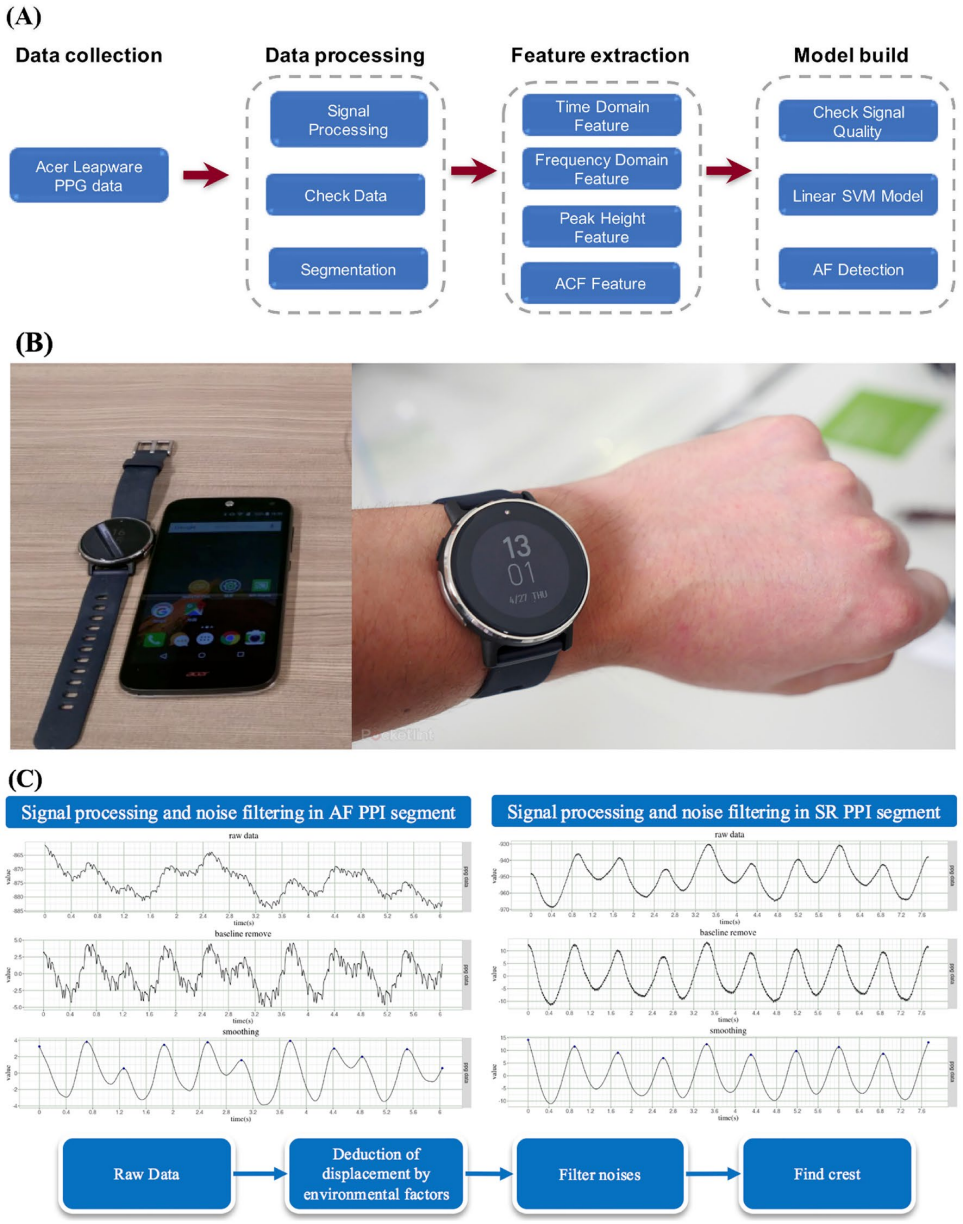
(**A**) Proposed framework of the photoplethysmogram (PPG) to detect atrial fibrillation (AF). (**B**) The wrist-worn smartwatch. (**C**) Signal preprocessing that includes baseline wandering reduction, denoising, and motion artifact detection.

We proposed an AF detection framework (Fig. 2A) based on the green-light PPG signal recorded by a wrist-worn smartwatch (Fig. 2B). The details of this method have been published in our previous studies^31,32^. Artifacts of the PPG signal caused by wrist movements are obstacles, as they make the measurement of the change in blood volume unreliable. Signal preprocessing, which included baseline wandering reduction, denoising, and motion artifact detection, was applied to the PPG signal before PPG feature extraction (Fig. 2C). After signal preprocessing, PPG features, including temporal, spectral, and morphological features, were extracted from 10, 25, 40, or 80 heartbeats of the split segments. The extracted PPG features were used as inputs in a support vector machine (SVM) for classification as AF or SR.

### Signal preprocess

Triaxial ACC and PPG signals were simultaneously recorded. The PPG signal was excluded if it reached the saturation value or if the proportion of missing data was > 5% (for example, a loss of 256 sample points in a 10-s segment for a sampling rate of 512 Hz). The ACC signal showed significant changes, indicating the detection of active wrist movements as accelerations at the wrist. The duration between two peaks of a PPG signal was defined as the pulse-to-pulse interval (PPI). A PPI segment was excluded if the corresponding x-axis, y-axis, and z-axis of the ACC signal satisfied the following condition: sd(accX) × 0.57 + sd(accY) × 0.58 + sd(accZ) × 0.57 > 23.12, where sd (x) is the standard deviation of the ACC signal over a 1-s period. Baseline wandering reduction and denoising were applied to the PPG signal using R package, R: smooth.spline function. We eliminated PPG baseline wandering using the smooth.spline function with degrees of freedom set at 10 and filtered the high-frequency noise using the smooth.spline function with degrees of freedom set at 100. Finally, the peak detection algorithm was applied to the processed PPG signal to extract PPI.

### Feature extraction

We detected the peak of the processed PPG signal using the R package, R:pracma: findpeaks function with the mini peak distance set at 50 and obtained the PPI sequence. The PPI sequence was then split into K segments such that each segment contained N (10, 25, 40, or 80) heartbeats. In this study, PPI was treated as a time-dependent random variable with a probability distribution f(x) and mean μ and variance σ^2^ as parameters. The proposed framework contained two types of features: the first calculated from one PPI segment with N heartbeats and the other from three PPI segments. The features extracted from one PPI segment included standard deviation (SD), root mean squared successive differences (RMSSD), Shannon entropy with bins of 10, 100, and 1000 (SE10, SE100, and SE1000, respectively), and maximum fast Fourier transform (FFT) frequency (MaxFFT). The features calculated from the three PPI segments were: average SD (SD3), SD of the RMSSD (RMSSD3), and SD of the maximum FFT frequency (MaxFFTSD3). For example, SD3 was calculated using the SD of the current PPI segment and two of the closest subsequent PPI segments. SD, RMSSD, and Shannon entropy were used to measure the variation in a PPI segment^33^. Higher SD and RMSSD values indicated higher variations in PPI. A continuous regular heartbeat with low variation must be present in PPI.

Shannon entropy is a quantity of uncertainty in a probability distribution. In this study, it represented the uncertainty in the PPI distribution. For N heartbeats in a PPI segment, the PPI distribution pdf(x) was estimated using bins B and then calculated as $-\sum_{b=1}^{B}{P}_{b}{\mathrm{log}(P}_{b})$, where ${P}_{b}$ is the histogram of the PPI in bin B. Generally, inclusion of a greater number of heartbeats allowed a more accurate estimation of the PPI distribution. The optimal number of bins required for PPI distribution estimation differed for the AF and SR cases. We used different numbers of bins to calculate Shannon entropy as features of the SVM to maintain the difference in uncertainty between the AF and SR cases.

In addition to the PPI-derived PPG features, we also extracted the morphological features of each PPI segment with N heartbeats. In this case, the peak height of the signal was treated as a random variable. Features including SD, RMSSD, and interquartile range (IQR), were calculated from the peak height and autocorrelation function (ACF) of the peak height of each PPI segment. The SD of the first difference was extracted from the ACF peak height for each PPI segment (Diff SD). The ACF measures time-dependent correlation of the peak height from N heartbeats.

To extract the features for other arrhythmias, the ACF of PPI, unimodality test p-value of a single segment, PPI SD, and odds PPI SD were considered. The following features were considered to extract the ‘regular abnormal’ heartbeat, for example, bigeminy heartbeat, from the VPCs or APCs: The N PPIs in one segment were used to calculate the lag 1 ACF function (Lag1ACF) and lag 2 ACF function (Lag2ACF). The unimodality test^34^ was performed to test the PPI frequency with a unique mode. The even (odd) PPI SD was calculated using the even (odd) numbers of PPIs in one segment.

### Classification and feature selection

Data collected from 116 patients included in our study group were used to train the SVM classification model and for feature selection. A PPI segment with N heartbeats was labeled as AF or SR, and the features from its own segment and two closest PPI segments were extracted. We used a five-fold cross-validation process for the training and feature selection. Specifically, signals from 116 patients were randomly split into five, and the model training procedure was then performed. The hyperparameters of the linear kernel for SVM and the features were selected with respect to the best-fit receiver operating characteristic (ROC) curve. The feature selection procedure excluded MaxFFT, but retained MaxFFTSD3. The hyperparameter cost of the linear kernel in the SVM was searched from 0 to 1.5, with a cost of 0.1 selected.

### Statistical analysis

Continuous variables are expressed as mean ± SD or median (IQR), as appropriate, for the data distribution. Categorical data are expressed as frequencies (percentages). In the univariate analysis of AF and SR data, chi-squared and Fisher’s exact tests were used to analyze categorical data. Student’s t-test was used to analyze continuous variables between the two sets of data. We used a univariate logistic regression model to estimate independent PPG features to differentiate between AF and SR. ROC curves were calculated to evaluate the diagnostic ability of the selected parameters. All statistical analyses were performed using R Statistics version 3.6.2 for Windows (R Foundation for Statistical Computing, Vienna, Austria).

## Data Availability

All data pertinent to this study is available within the article. Additional data not available due to ethical and legal restriction.
